# Supplementary figures and images for: Benchmarking ultra-high molecular weight DNA preservation methods for long-read and long-range sequencing
Source: Gigascience. 2022 Aug 10;11:giac068. doi: 10.1093/gigascience/giac068 (PMC9364683; doi:10.1093/gigascience/giac068)

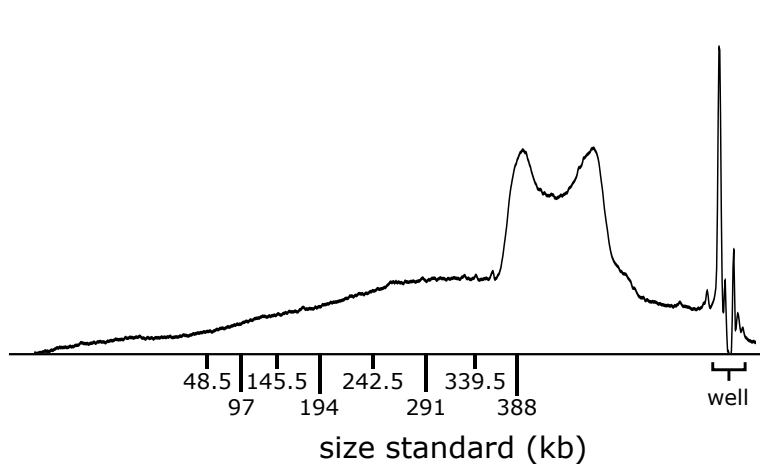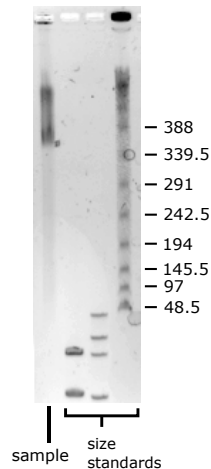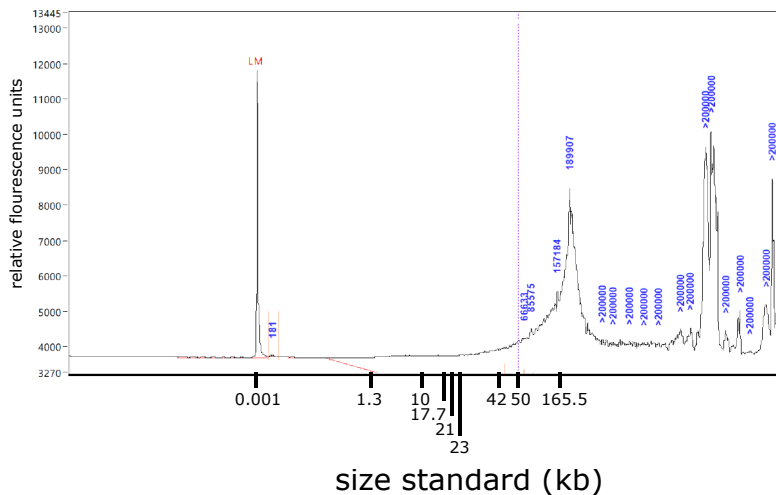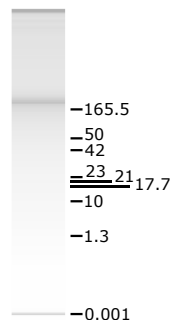

Supplement: giac068_Supplemental_Figures_and_Tables [file giac068_supplemental_figures_and_tables.zip › Fig.S1_supplemental material.pdf]

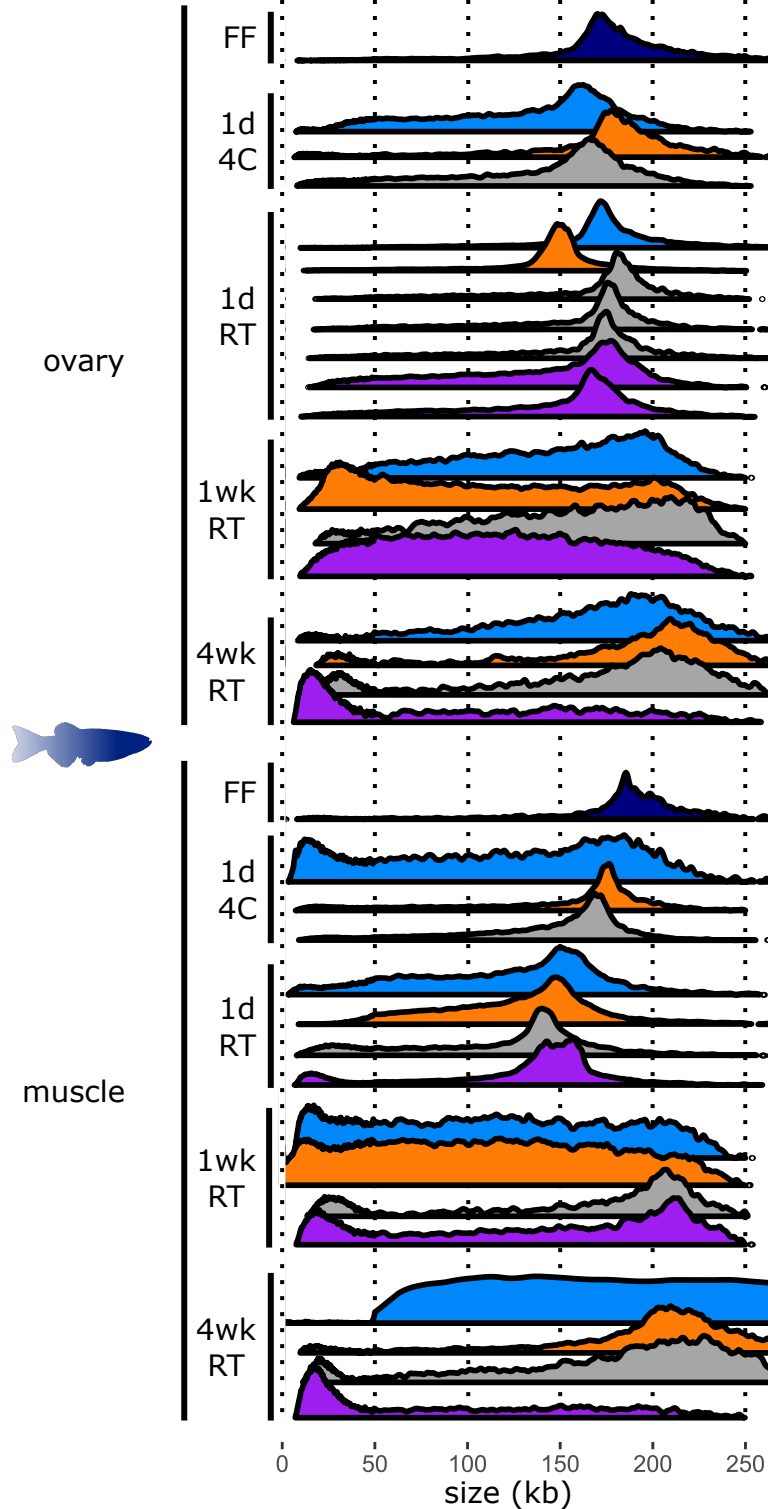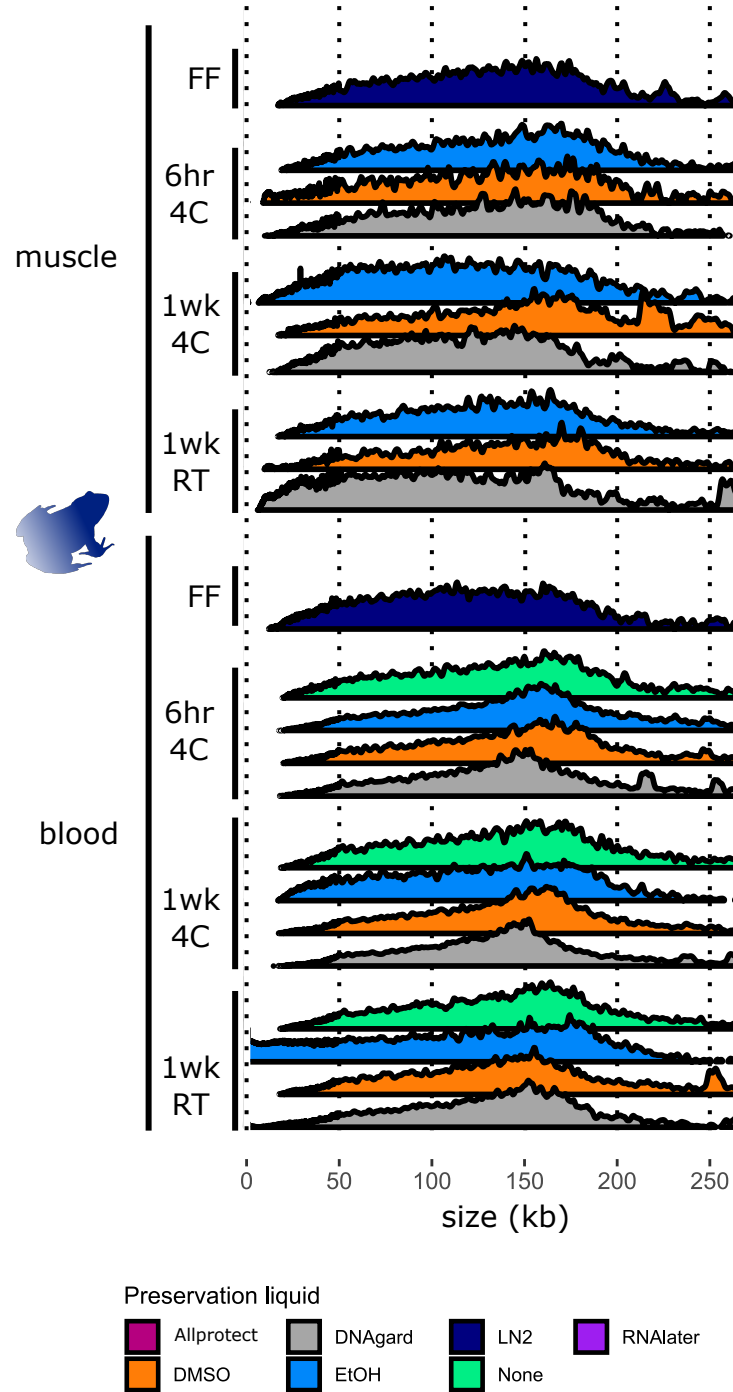

Supplement: giac068_Supplemental_Figures_and_Tables [file giac068_supplemental_figures_and_tables.zip › Fig.S2_supplemental material.pdf]

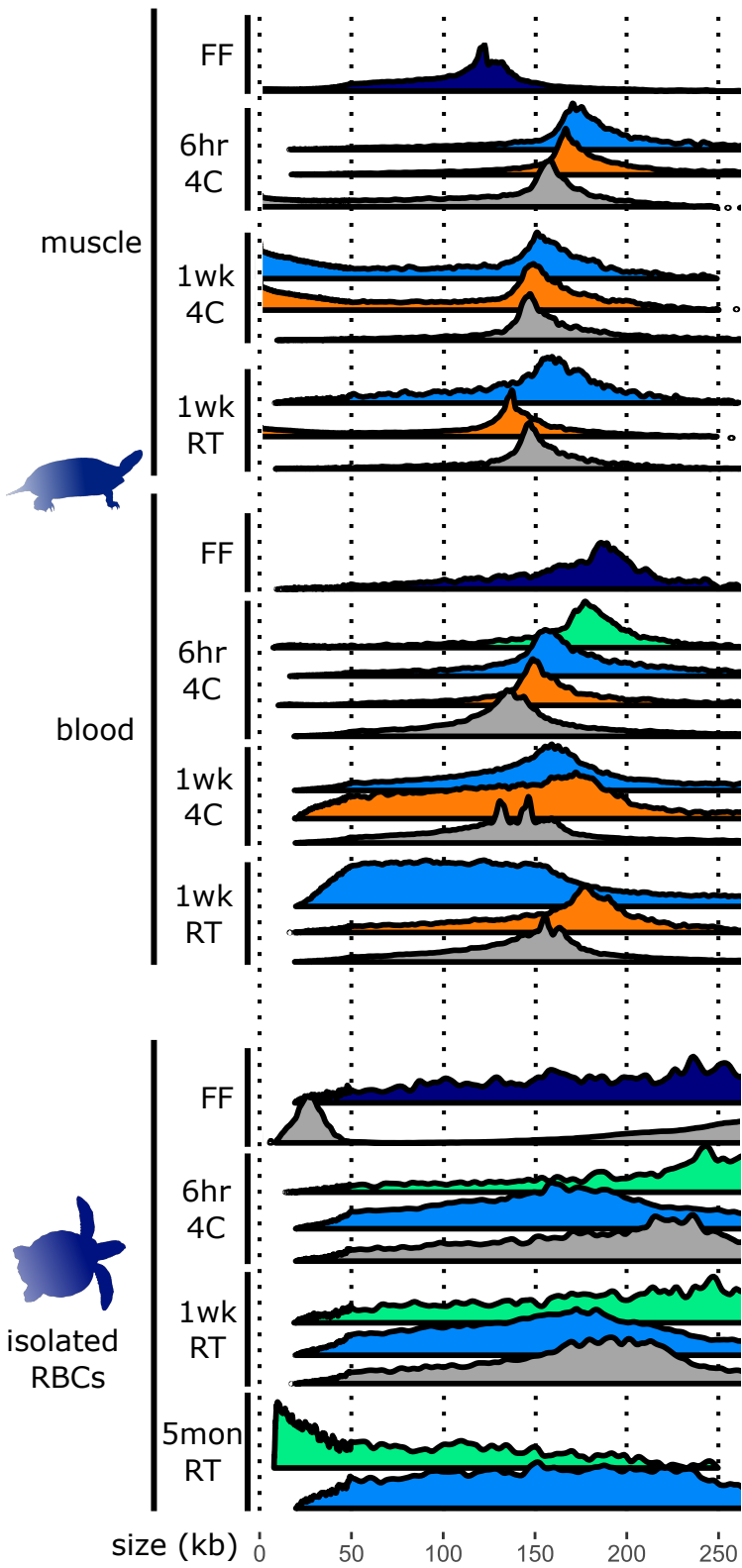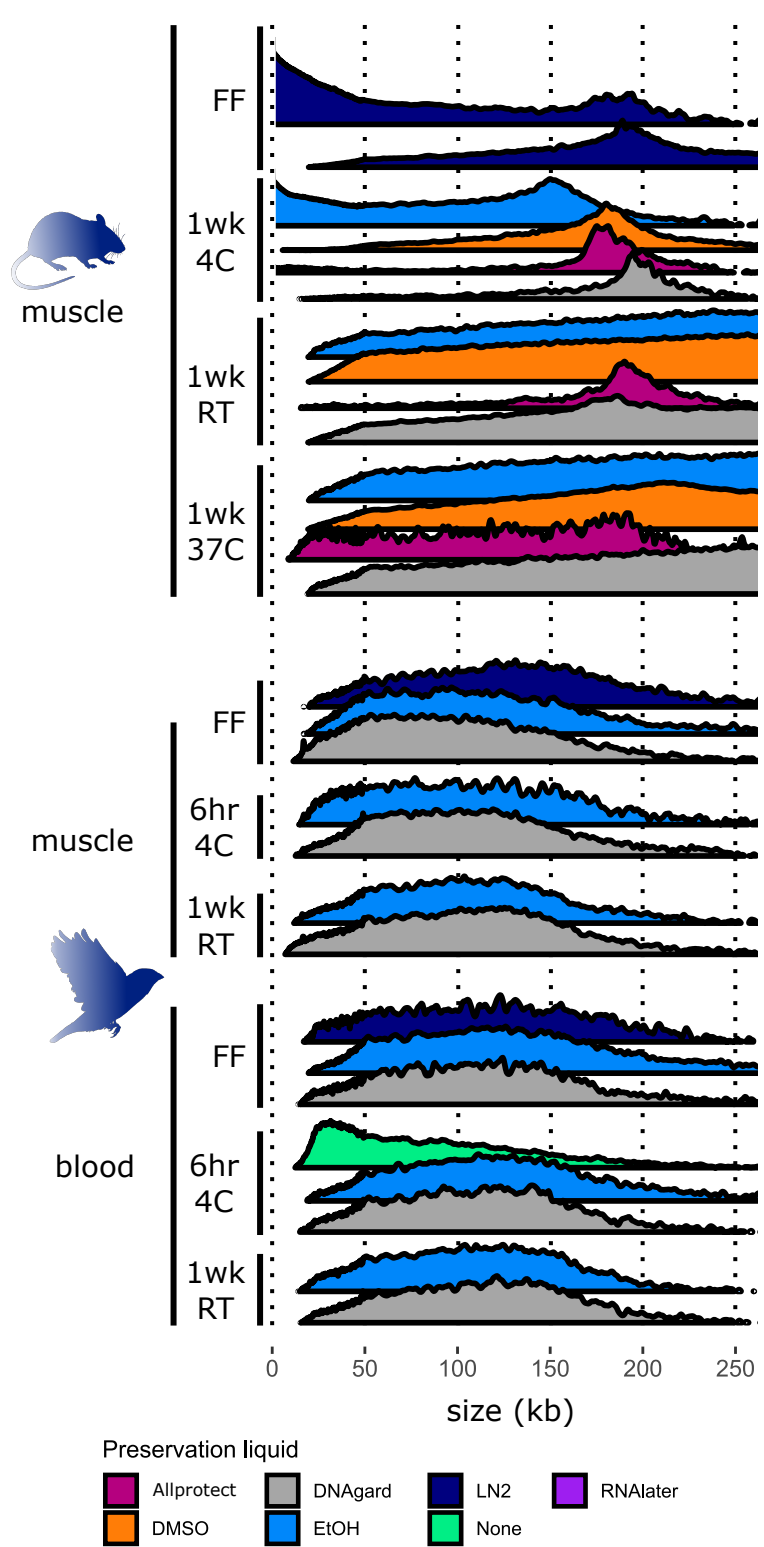

Supplement: giac068_Supplemental_Figures_and_Tables [file giac068_supplemental_figures_and_tables.zip › Fig.S3_supplemental material.pdf]

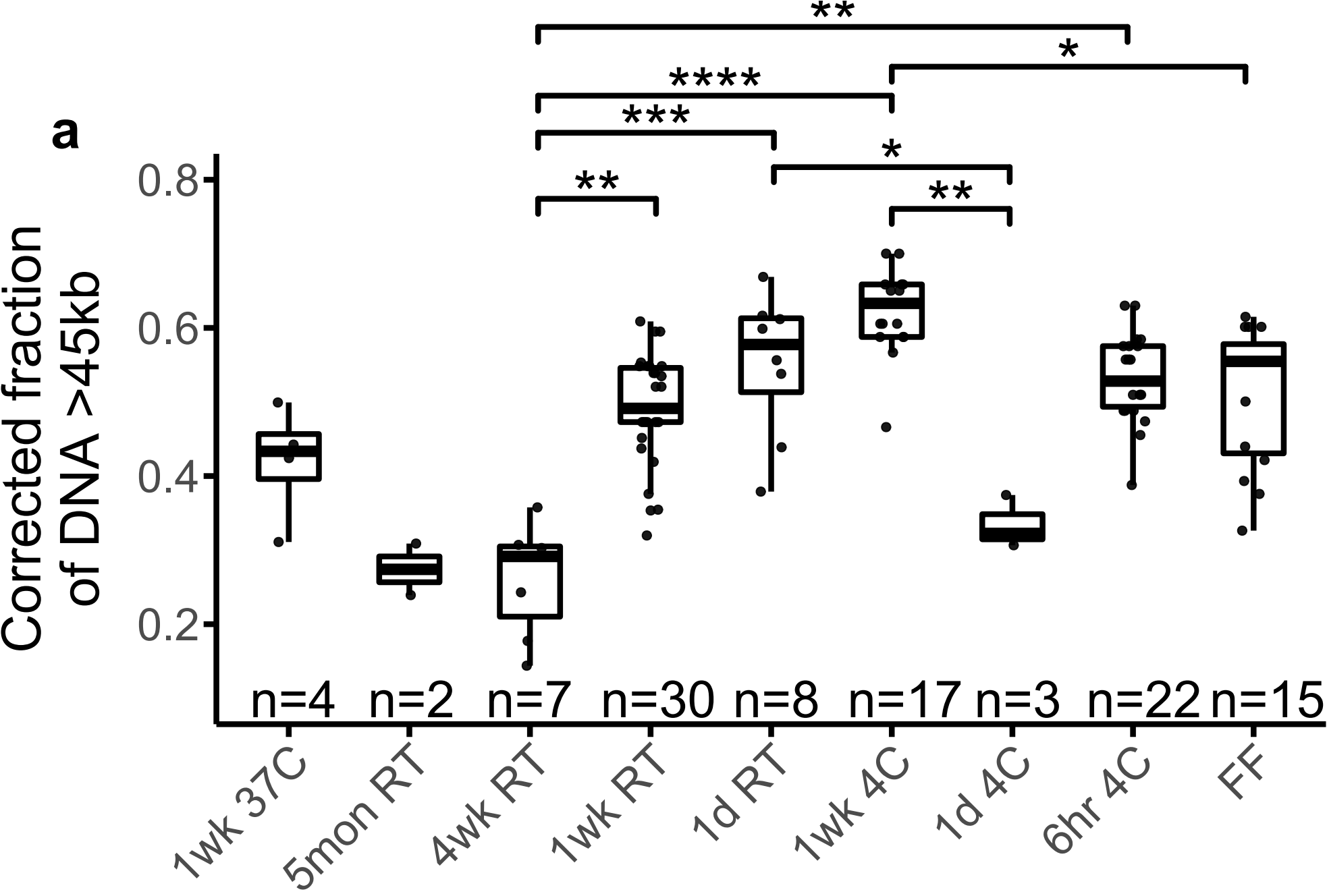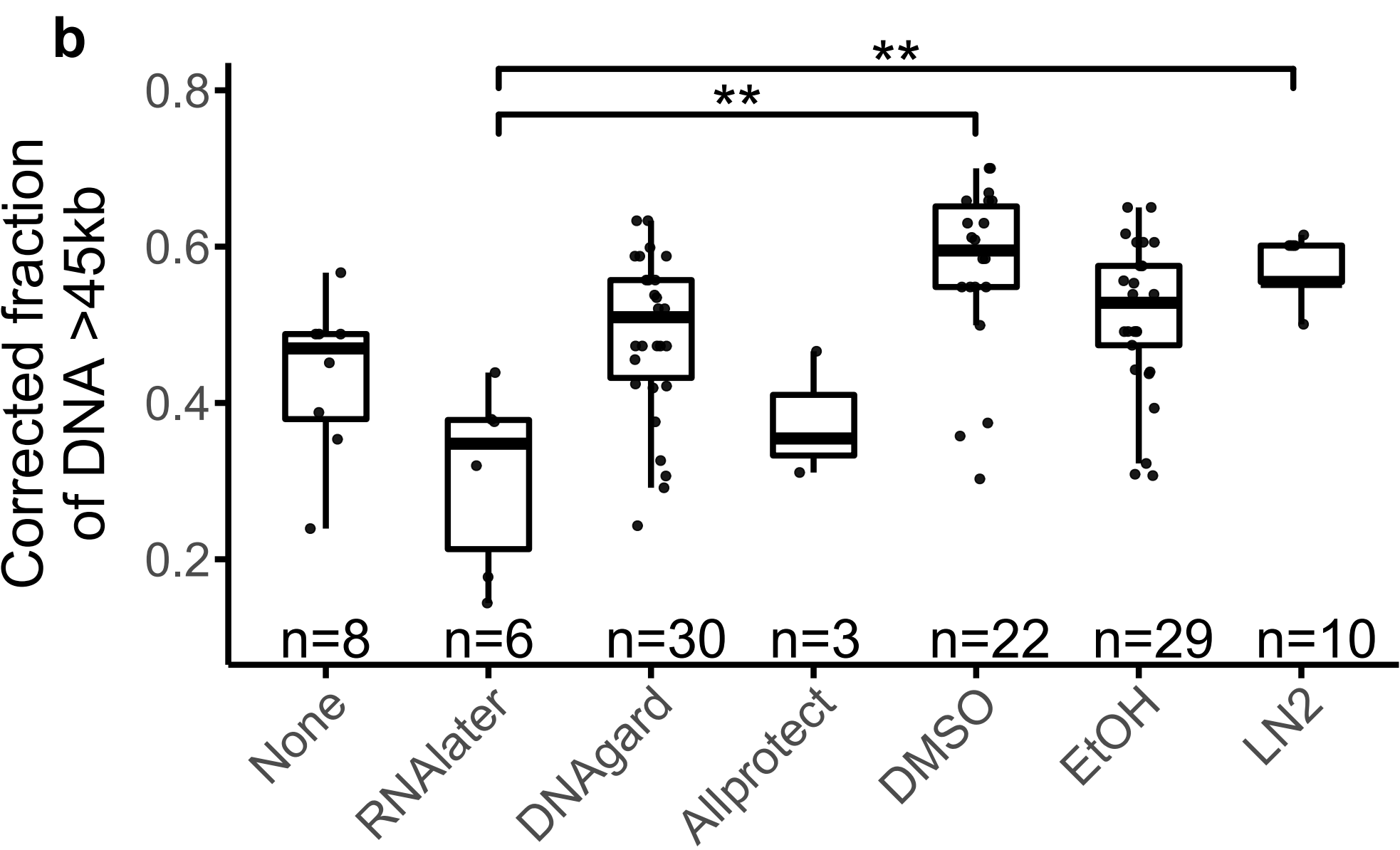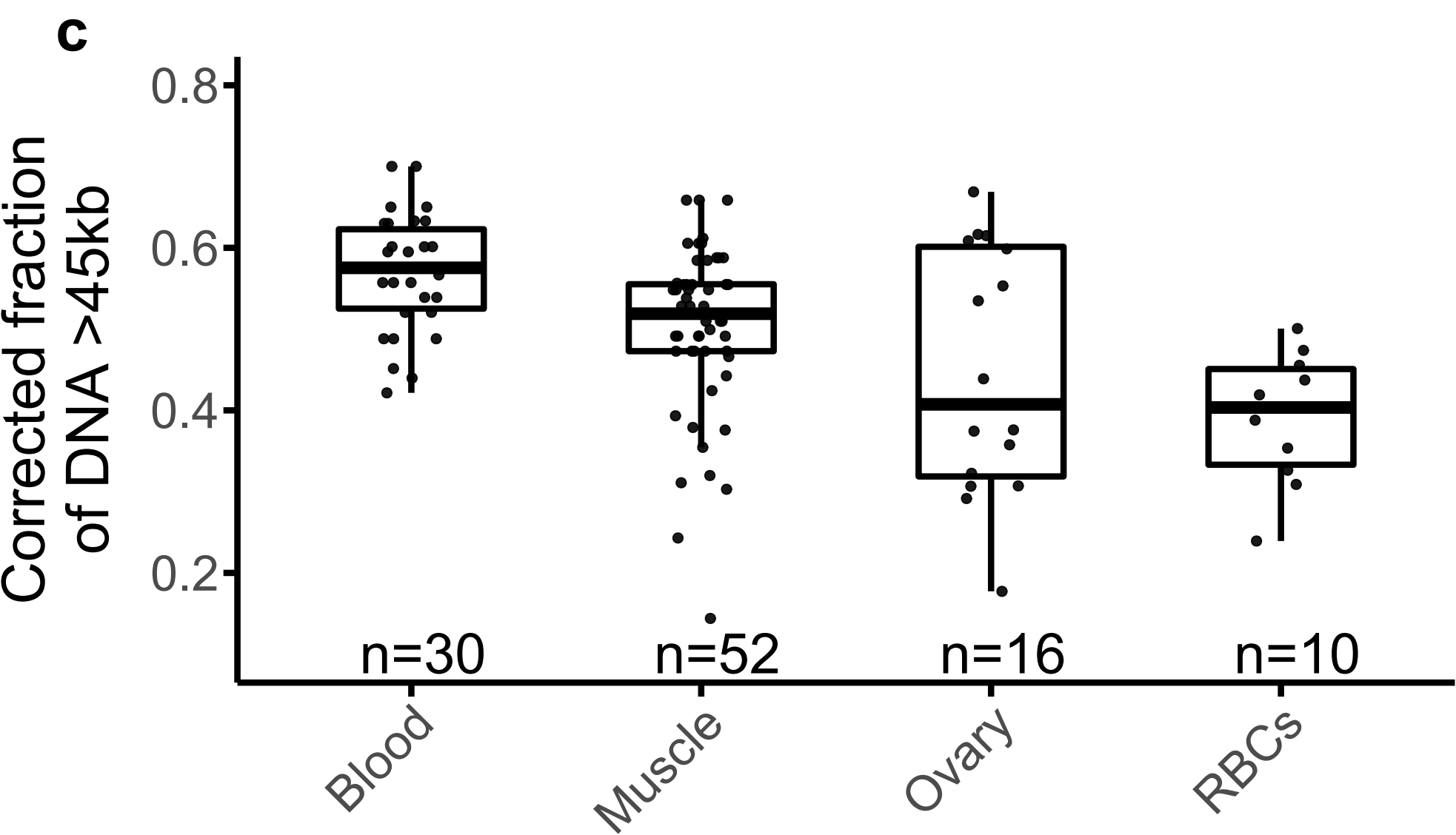

Supplement: giac068_Supplemental_Figures_and_Tables [file giac068_supplemental_figures_and_tables.zip › Fig.S4_supplemental material.pdf]

**a**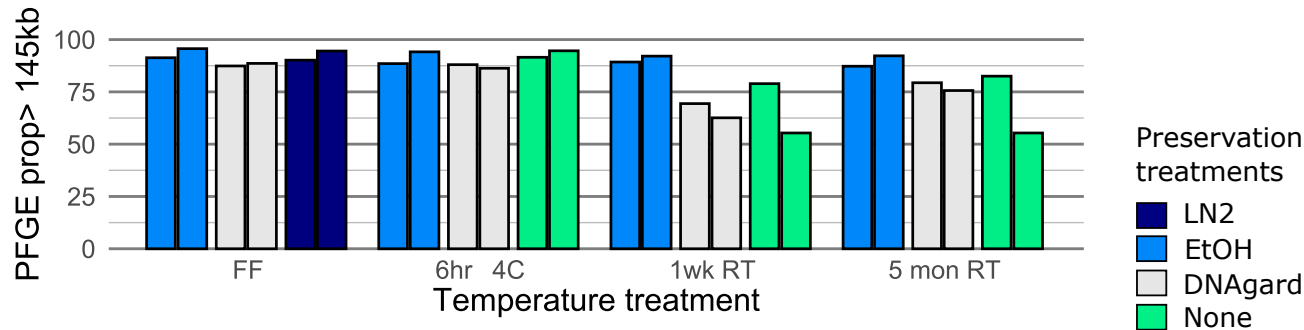**b**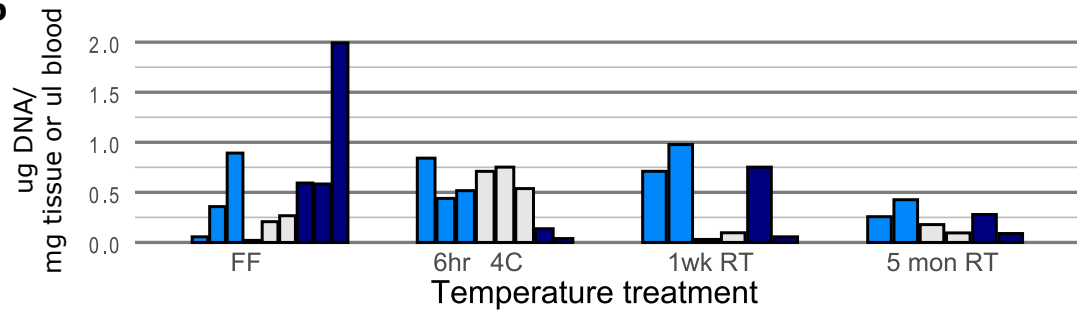

Supplement: giac068_Supplemental_Figures_and_Tables [file giac068_supplemental_figures_and_tables.zip › Fig.S5_supplemental material.pdf]
