# Supplementary material for: Benchmarking ultra-high molecular weight DNA preservation methods for long-read and long-range sequencing
Source: Gigascience. 2022 Aug 10;11:giac068. doi: 10.1093/gigascience/giac068 (PMC9364683; doi:10.1093/gigascience/giac068)
Supplement: giac068_Response_to_Reviewer_Comments_Original_Submission [file giac068_response_to_reviewer_comments_original_submission.pdf]

# Author's Response To Reviewer Comments

Close

Dear Dr. Hans Zauner,

Thank you for the opportunity to revise and resubmit our article titled “Benchmarking ultra-high molecular weight DNA preservation methods for long-read and long-range sequencing” to GigaScience. We greatly appreciate the effort put forth by you and the reviewers in providing feedback on our manuscript. The comments we received are insightful suggestions that we feel have improved the manuscript. We have been able to incorporate changes based on these contributions, as highlighted below.

Comments from Reviewer 1:
R. 1, Comment 1: In figure 2 the size distribution of DNA fragments is visualized from the different experiments. Most of the fragment distributions look like I would have expected them based on the work we did in the article cited as nr 25 in the reference list. However the muscle tissue from rats and the blood samples from the mouse and the frog indicates that there may be a misinterpretation in the article regarding the actual size distribution of fragments which needs to be looked in to.
Response: The following comments indicate two major limitations to this methodology: the interpretable range of fragment sizes, and streaking artifacts. In the article we endeavor to interpret these results within the bounds of those limitations. We’ve added additional text and made figure modifications, detailed below, to clarify these caveats.

R. 1, Comment 2: Starting with the mouse plots and especially the muscle one. There must either have been a physical shearing event that drastically reduced the size of DNA (using the terminology from ref 25 this would mean that physical shearing generated a characteristic fragment length of approximately 300-400 kb), or the lack of a sharp slope on the rightmost side of the ridgeline plot is due to the way the image was processed. All other animals got a peak on the rightmost side of the ridgeline plot and the agarose plug should, based on the referenced methods paper [7], generate megabase sized fragments which far exceed the size of the scale used in figure 2. I would presume these larger fragments would get stuck in or near the well which makes it easy to accidentally cut them out when doing the image analysis step which may explain their absence in the mouse samples. This leads me to the conclusion that the article is well designed to capture the impact of chemical shearing caused by different preservation methods but would benefit from evaluating whatever figure 2 properly covers the actual size distribution of fragments or only covers the portion of DNA fragments small enough to actually form bands on the PFGE gel with a substantial part of the DNA stuck in or near the well.
Response: This is a correct indication of the limits of visualizing DNA fragment length distributions with current technologies, only fragments that fall within a given range can be viewed at once with any reasonable resolution. We targeted a range of fragment sizes that includes ideal sizes for long-read and long-range sequencing while also giving indications of degradation in the smaller end of the range. Fragments outside the target range can not be reliably interpreted. In PFGE, DNA fragments larger than the target range can be stuck in or near the well (right side of the plots). Unfortunately, in the well they then become indistinguishable from bright reflections off the edges of the well and are further obscured when streaking is present. We attempted early on to score samples for presence/absence of signal in or near the well, but found this to not be repeatable or informative using the gel images. Thus, we chose to omit the well itself and the space immediately around it from interpretation, from measurements for statistical testing, and from Fig. 2. We only interpret the portion of the PFGE gel where bands can form and where the standard can give us some indication of fragment size. The interpretable range of fragment sizes in these gels still offers important indicators of sample quality.
For clarity, the new version of Fig. 2 includes the well in each plot profile. The well peaks are cropped where they exceed the peak brightness of the rest of the lane. We do not consider the well brightness to be a reliable indicator of sample quality, but recognize that some readers may want to see the full pattern.
The following has also been added to the Fig. 2 legend: “Fluorescent stained DNA fragments are drawn with an electric current from the well at the right towards the left. Smaller fragments generally travel farther than larger fragments. The fragments that greatly exceed the targeted size range remain in the

well and can not be reliably interpreted.”
“The well brightness is cropped where it exceeds the brightness of the rest of the gel lane.”
We have also added this text to the discussion section of the manuscript: “Additionally, we are only able to visualize DNA fragment size distributions within a certain range of sizes (approx. 40–400 kb for PFGE, 1.3–165 kb for FEMTO). Though we have targeted a size range that includes both ideal fragment sizes for long-read sequencing and fragments of lower molecular weight that may indicate degradation, fragments outside this range are not measured here.”

R. 1, Comment 3: The frog plot is a good example of how this may influence our interpretation of the ridgeline plots. If the extraction method generate high-quality DNA concentrated in the 300-400 kb range then there must be something very special with the frog DNA from blood as there is a continuous increase in the brightness all the way to the edge of the image. This implies that the sample contains a high amount of much larger DNA fragments than the other samples. I find this rather unlikely and if I saw this in my own data I would assume that we had a lot of very large DNA fragments that are out of scale for the gel electrophoresis but that in the case for the frog blood samples many of these fragments have been chemically sheared creating the "smeared" pattern we see in figure 2.
Response: Yes, the frog blood especially exhibits a “streaking” pattern in the gel where there is a strip of continuous brightness in the lane. This is another reason why we do not attempt to interpret the gel above where bands form in the lane. Before our initial submission, we performed a repeat run of the PFGE gels with streaking, but they produced identical results with streaks still present. Samples with this streaking pattern have performed well in past sequencing efforts, and it’s generally thought to be an indicator of high quality samples. However, barring more conclusive testing of this pattern, we do not attribute streaking as an indicator of quality in this manuscript.
We have added this section to the figure caption: “DNA fragments with lengths longer or shorter than peaks of the size standard can not be reliably interpreted due to lack of size reference and artifacts of gel electrophoresis as well as limitations of any type of gel electrophoresis to correctly size megabase-length fragments.”

R. 1, Comment 4: Dryad DOI doesn't work for me.
Response: It is possible that the reviewer was attempting to open the link provided in the cover letter, which has formatting mistakes related to the uploading process. A correct link was supplied to Dr. Zauner. Apologies for this inconvenience. Here is the correct and updated Dryad download link for reviewers: https://datadryad.org/stash/share/uHgVucrNICiMT-Y92O4M4Km3S4DyK3UJFA3qMJEbm4M

R. 1, Comment 5: Figure 1 - The meaning of x3 and x2 for the turtle should be described in the caption.
Response: The Figure 1 caption now has the added sentence “For the sea turtle samples, cells with numbers (x2 or x3) indicate conditions where samples from more than one individual were processed for comparison.”

R. 1, Comment 6: Figure 2 - Having the scale indicator (48.5. 145.5 etc) at the top as well as the bottom of each column would make it quicker to estimate the distribution of samples.
Response: Agreed. This has been added to Figure 2.

R. 1, Comment 7: The article completely omits Nanopore sequencing, is there a specific reason for why lessons here are not applicable to ONT?
Response: Nanopore equipment and expertise were not available at the time of this study, but will be part of further testing. We generally expect the same indicators of sample quality to correlate with successful sequencing in Nanopore sequencing as with other technologies, though it is not explicitly tested here. We’ve added a mention of Oxford Nanopore to the list of relevant technologies in the introduction to clarify: “Long-reads (generally > 10 kb; e.g. Pacific Biosciences or Oxford Nanopore), long-range molecules (generally > 50 kb; e.g. 10X Genomics linked reads), or optical mapping (> 150 kb; e.g. Bionano Genomics), and Hi-C proximity ligation (> 1 Mb; e.g. Arima Genomics) can span repeats thousands of base pairs in length [4], greatly improving assembly outcomes.”

R. 1, Comment 8: There is a very interesting paragraph starting with "The ambient temperature of the intended collecting locality should be a major consideration in
planning field collections for high-quality samples. Here we test a limited number of samples at 37°C to". Even if the results were very poor information about the failed conditions would be appreciated. What tissues/animals did you use, did you do any preservation at all for the samples and did you measure the fragment length distribution anyway? Simply put, even if the DNA was useless for long read sequencing it is an interesting data point for the dynamics of DNA degradation and a valuable lesson for planning sampling in warm climates.

Response: The “limited number of samples” refers to the four mouse muscle samples reported with the rest of our results. No further samples were tested at 37°C in this study. We’ve modified that sentence for clarity as follows: “Here we test a limited number of samples at 37°C to resemble fieldwork conditions in warmer climates, resulting in no retention of workable amounts of uHMW DNA in any of these samples (4 mouse muscle samples; Fig. 2).”

Comments from Reviewer 2:
R. 2, Comment 1: Although the effectiveness of the tissue/preservative combination was only tested with the preparation of long range libraries, it would have been useful to select one or two cases for long range sequencing (PacBio or Oxford Nanopore) to explore the impact of the different QC parameters measured in this study.
Response: We agree that testing samples on long-read sequencing platforms would have been very useful. Unfortunately, the expense of long-read sequencing was prohibitive at the time. We do find that the results align generally with the experience of the Vertebrate Genomes Project. See response to the related comment #4 below for further details.

R. 2, Comment 2 (in text): space between quantity and unit symbol
Response: This change has been applied to the text.

R. 2, Comment 3 (in text): such as used twice in a sentence. please edit
Response: This sentence has been revised.

R. 2, Comment 4 (in text): Your work is a great contribution to the genomics field!
However, DNA integrity and optimal QC parameters (Absorbance ratios at 260/230, 260/280, double stranded DNA proportion from a total gDNA prep) are not always predictors for Long Read Sequencing success. I am aware of the high cost that you would face if all these samples were sequenced, even one flowcell/sample in minION, could cost a little fortune. But it would be fantastic if you could please indicate if any of the sample/preservation combinations showed consistent good LRSeqeuncing results.
Response: We concur that simply checking the DNA integrity and yield as QC parameters might not give a definitive indicator of sequencing success with long reads. Moreover, this particular paper has been concentrating on Vertebrata, a relatively small taxonomic group as compared to Arthropoda, Planta, etc.

Additionally, as new reagents for DNA preservation are constantly emerging on the market (e.g. Allprotect), sample collection committees within VGP, EBP, ERGA and other large reference genome sequencing initiatives will continue monitoring and studying the impact of those products on the sequencing outcome, but those would be subjects of other studies, publications, and public guidelines.

From the experience of several large PacBio sequencing providers, including the facilities involved in this publication, we know that the chemical purity of the HMW-DNA sample is at least as important as the molecule integrity. There is, however, no single definitive analytical parameter that has ever been defined for predicting the long-read sequencing outcome based, or for detection of any carry-over contaminants or significance to sequencing success. For this reason, chemical purity parameters were not within the scope of this manuscript, though they likely carry influence outside the variables manipulated in this study.

We have instead concentrated this study on the low-hanging fruit of integrity of HMW-DNA molecules and DNA yield, both of which robustly and intuitively influence sequencing success. From that point of view, our smaller sequencing tests and general experience outside the scope of this manuscript corroborate the results presented in the study. Two of these smaller tests are detailed below.

Recent test 1: Flash-frozen vs. EtOH-preserved reptile tissue
We compared the length of uHMW gDNA and performance on Bionano and PacBio continuous long reads of snap-frozen and EtOH-preserved nucleated blood from reptiles and found no significant changes in performance in Bionano molecule size and PacBio CLR sequencing subread length. The average length of unfiltered Bionano reads was longer when gDNA was extracted from flash-frozen tissue, though both treatments still returned results in an acceptable range. In general, Bionano optical mapping was working reliably for gDNA from EtOH-preserved tissues.
Some of the quality specifications are shown in the table in the attached "Response to reviewers" document.

Recent test 2:
We used mammalian kidney tissue that had been stored in Allprotect recently for PacBio HiFi and Hi-C

(ARIMA protocol). Right after tissue extraction, the tissue was soaked in Allprotect at room temperature overnight, stored for about 1 year at -20C degrees, then shipped, and finally stored at -80 degrees before sequencing. The sample showed no indication of any contaminants on a Nanodrop spectra readout. Please see the PFGE gel image and summarized sequencing results in the attached "Response to reviewers" document.

R. 2, Comment 5 (in text): Did you load the same amount of DNA per PFGE lane for all samples?

Did you heat up the DNA sample + loading buffer before loading? 1-2 min at 65C followed by cooling at room temperature ~ 5 min before loading helps to prevent clumping.

Did you run a slice of each plug as a control for the DNA manipulation factor that could cause fragmentation while extracting the DNA from the plug?
Response:
Yes, we loaded approximately 100 ng of DNA per well.

No, we did not heat DNA and loading buffer prior to loading. DNA, loading buffer, and TE buffer were kept at room temperature before mixing and then loading. DNA was loaded after at least one week at room temperature, which allows for homogenization of the sample and increases hydration of the DNA molecules in the sample. Although the original HMW DNA samples are often viscous, the addition of TE buffer and loading buffer dilutes them to the point that we find clumping is reduced.

No, each plug was carried through entirely for DNA extraction. Digesting the entire plug was integral to comparing DNA yield. The process to extract DNA from the plug is quite gentle (slow shaking for lysing and washing steps, Agarase digestion, drop dialysis) and the plug also protects the DNA.

R. 2, Comment 6 (in text): The picture shows embedded nuclei with or without crosslinker, but the same could be done with the extracted DNA from plugs
Response:
We absolutely agree that the conventional field-inversion PFGE instruments (e.g. BioRad CHEF) are much better suited to resolve sub-megabase size DNA fragments from in situ extractions. However, this specialized equipment is currently much less common as compared to a cheaper (albeit less precise) Pippin Pulse system from SAGE which is widely used at sequencing facilities.

R. 2, Comment 7 (in text): Perhaps analyzing a sample in a standard agarose gel might help evaluate fragmentation < 20 kbp.
Response: This is certainly something that could be incorporated into future testing as another metric of the smaller fragment sizes, perhaps as a cheaper alternative to FEMTO. Unfortunately, more testing at this point on the same extractions would not be useful; they are now several years old. Our subset of samples tested on the FEMTO Pulse system give a detailed perspective on <20 kb fragments.

R. 2, Comment 8 (in text): It might help to describe briefly how Arima prepared the nucleated blood Hi-C libraries, especially the samples preserved in DNAgard. I think this solution does contain an inhibitor of the crosslinking reaction, most likely a free amino group (from Tris buffer, for example). The Dovetail Genomics Omni-C kit protocol dilutes the nucleated blood preserved in EDTA tubes in 1 mL 1X PBS buffer and collects the cells by centrifugation before the crosslinking steps. Sorry I don't have access to the Arima protocol document to make a more informed comment.
Response: Our procedure for nucleated blood in a solution like ethanol (or DNAgard) is to pellet the cells, remove the supernatant, wash with 1X PBS containing 1% FBS, and then carry the washed pelleted cells into crosslinking and then Arima-HiC. Given our washing procedure, it seems less likely (although still possible) that residual tris is inhibiting the crosslinking reaction. Interestingly, DNAgard is a proprietary solution originally developed by Biomatrica, and so we were not able to find any resource that pertains to what the solution is actually composed of.
We’ve added a citation of the protocol document number and this note to the methods: “Briefly, standard protocol for nucleated blood in a solution like EtOH or DNAgard is to pellet the cells, remove the supernatant, wash with 1X phosphate buffered saline solution containing 1% Fetal Bovine Serum, and then carry the washed pelleted cells into crosslinking and then Arima-HiC.”, and this to the discussion: “Though our washing protocol should minimize its effect, it is also possible that some unknown aspect of the DNAgard treatment of cells inhibited the crosslinking reaction, and Hi-C of unfixed cells would be expected to have low signal and high noise similar to degraded DNA.”

Additional clarifications:

INSDC submission - We have removed the Hi-C sequence reads from the manuscript's associated Dryad repository and are in the process of uploading them to the publicly accessible Sequence Read Archive.
Analysis scripts - Two commented scripts have been added to the manuscript's associated Dryad repository. One contains statistical analysis of DNA yield and fragment length reported in the manuscript, and the other has basic bioinformatics and calculations based on the Hi-C reads reported in the manuscript.
The data availability section of the manuscript has also been updated to reflect these changes.

Please feel free to notify me of any further comments or questions. We look forward to your response.

Thank you again for your hard work and for this opportunity.

Sincerely,

Olivier Fedrigo, Ph.D.
Director
Vertebrate Genome Laboratory

Close
