# Supplementary material for: Benchmarking ultra-high molecular weight DNA preservation methods for long-read and long-range sequencing
Source: Gigascience. 2022 Aug 10;11:giac068. doi: 10.1093/gigascience/giac068 (PMC9364683; doi:10.1093/gigascience/giac068)
Supplement: giac068_Response_to_Reviewer_Comments_Revision_1 [file giac068_response_to_reviewer_comments_revision_1.pdf]

Author's Response To Reviewer Comments

Close

Dear Dr. Hans Zauner,

Thank you for overseeing the final steps of this process and for bringing these needed changes to our attention. We have adjusted the manuscript as follows:

1) Data citation.
The GigaDB dataset has been appended to our reference list and cited in the data availability section.

2) Accession numbers.
We have added the BioProject and BioSample accession numbers from DDBJ to the data availability section.

3) Supplemental file descriptions.
We have added descriptive titles and legends for each referenced supplemental file to the end of the manuscript document.

4) Abstract.
We have revised the abstract in the “Backgrounds”, “Results”, “Conclusions” structure, as requested.

5) ORCIDs.
We have added a list of ORCIDs to the title page.

Please feel free to reach out if any further adjustments are necessary before publication. Thank you again for your oversight and hard work.

Sincerely,
Dahn, et al.

Close
