# Supplementary material for: Benchmarking ultra-high molecular weight DNA preservation methods for long-read and long-range sequencing
Source: Gigascience. 2022 Aug 10;11:giac068. doi: 10.1093/gigascience/giac068 (PMC9364683; doi:10.1093/gigascience/giac068)
Supplement: giac068_Reviewer_1_Report_Original_Submission — Tomas Sigvard KlingstrÃ¶m -- 10/26/2021 Reviewed [file giac068_reviewer_1_report_original_submission.pdf]

Reviewer Report

Title: Benchmarking ultra-high molecular weight DNA preservation methods for long-read and long-range sequencing

Version: Original Submission **Date:** 10/26/2021

Reviewer name: Tomas Sigvard KlingstrÃ¶m

Reviewer Comments to Author:

As a researcher who may occasionally use long read sequencing technique for projects it is immensely helpful to get an insight into the experience accumulated through work related to the Vertebrate Genomes Project (VGP). My personal research interest on the subject is more on understanding why and how DNA fragment during DNA extraction. Due to my work in that area I have one key question regarding the interpretation of the data presented in figure 2 and then a number of suggestions for minor edits. The answer on how to interpret figure 2 may require some minor edits but the article is regardless of this a welcome addition to what we know about good practices for DNA extraction generating ultra high molecular weight DNA. It should also be noted that the DOI link to Data Dryad seems broken and I have therefore not look at the supplementary material.
In figure 2 the size distribution of DNA fragments is visualized from the different experiments. Most of the fragment distributions look like I would have expected them based on the work we did in the article cited as nr 25 in the reference list. However the muscle tissue from rats and the blood samples from the mouse and the frog indicates that there may be a misinterpretation in the article regarding the actual size distribution of fragments which needs to be looked in to.
Starting with the mouse plots and especially the muscle one. There must either have been a physical shearing event that drastically reduced the size of DNA (using the terminology from ref 25 this would mean that physical shearing generated a characteristic fragment length of approximately 300-400 kb), or the lack of a sharp slope on the rightmost side of the ridgeline plot is due to the way the image was processed. All other animals got a peak on the rightmost side of the ridgeline plot and the agarose plug should, based on the referenced methods paper [7], generate megabase sized fragments which far exceed the size of the scale used in figure 2. I would presume these larger fragments would get stuck in or near the well which makes it easy to accidentally cut them out when doing the image analysis step which may explain their absence in the mouse samples. This leads me to the conclusion that the article is well designed to capture the impact of chemical shearing caused by different preservation methods but would benefit from evaluating whatever figure 2 properly covers the actual size distribution of fragments or only covers the portion of DNA fragments small enough to actually form bands on the PFGE gel with a substantial part of the DNA stuck in or near the well.
The frog plot is a good example of how this may influence our interpretation of the ridgeline plots. If the extraction method generate high-quality DNA concentrated in the 300-400 kb range then there must be something very special with the frog DNA from blood as there is a continuous increase in the brightness all the way to the edge of the image. This implies that the sample contains a high amount of much larger DNA fragments than the other samples. I find this rather unlikely and if I saw this in my own data I would

assume that we had a lot of very large DNA fragments that are out of scale for the gel electrophoresis but that in the case for the frog blood samples many of these fragments have been chemically sheared creating the "smeared" pattern we see in figure 2.

Minor edits and comments:

Dryad DOI doesn't work for me.

Figure 1 - The meaning of x3 and x2 for the turtle should be described in the caption.

Figure 2 - Having the scale indicator (48.5. 145.5 etc) at the top as well as the bottom of each column would make it quicker to estimate the distribution of samples.

The article completely omits Nanopore sequencing, is there a specific reason for why lessons here are not applicable to ONT?

There is a very interesting paragraph starting with "The ambient temperature of the intended collecting locality should be a major consideration in
planning field collections for high-quality samples. Here we test a limited number of samples at 37Â°C to". Even if the results were very poor information about the failed conditions would be appreciated. What tissues/animals did you use, did you do any preservation at all for the samples and did you measure the fragment length distribution anyway? Simply put, even if the DNA was useless for long read sequencing it is an interesting data point for the dynamics of DNA degradation and a valuable lesson for planning sampling in warm climates.

Methods

Are the methods appropriate to the aims of the study, are they well described, and are necessary controls included? Choose an item.

Conclusions

Are the conclusions adequately supported by the data shown? Choose an item.

Reporting Standards

Does the manuscript adhere to the journal's guidelines on minimum standards of reporting? Choose an item.

Choose an item.

Statistics

Are you able to assess all statistics in the manuscript, including the appropriateness of statistical tests used? Choose an item.

Quality of Written English

Please indicate the quality of language in the manuscript: Choose an item.

Declaration of Competing Interests

Please complete a declaration of competing interests, considering the following questions:

- Have you in the past five years received reimbursements, fees, funding, or salary from an organisation that may in any way gain or lose financially from the publication of this manuscript, either now or in the future?
- Do you hold any stocks or shares in an organisation that may in any way gain or lose financially from the publication of this manuscript, either now or in the future?
- Do you hold or are you currently applying for any patents relating to the content of the manuscript?
- Have you received reimbursements, fees, funding, or salary from an organization that holds or has applied for patents relating to the content of the manuscript?
- Do you have any other financial competing interests?
- Do you have any non-financial competing interests in relation to this paper?

If you can answer no to all of the above, write 'I declare that I have no competing interests' below. If your reply is yes to any, please give details below.

I have collaborated with Olga Vinnere Pettersson on an article cited in this article. That article is mildly related but takes a much more theoretical approach while this article takes a hands on approach resolving many questions of interest.

I agree to the open peer review policy of the journal. I understand that my name will be included on my report to the authors and, if the manuscript is accepted for publication, my named report including any attachments I upload will be posted on the website along with the authors' responses. I agree for my report to be made available under an Open Access Creative Commons CC-BY license (http://creativecommons.org/licenses/by/4.0/). I understand that any comments which I do not wish to be included in my named report can be included as confidential comments to the editors, which will not be published.

Choose an item.

To further support our reviewers, we have joined with Publons, where you can gain additional credit to further highlight your hard work (see: https://publons.com/journal/530/gigascience). On publication of this paper, your review will be automatically added to Publons, you can then choose whether or not to claim your Publons credit. I understand this statement.

Yes Choose an item.
